# Smoking and Female Sex: Independent Predictors of Human Vascular Smooth Muscle Cells Stiffening

**DOI:** 10.1371/journal.pone.0145062

**Published:** 2015-12-14

**Authors:** Carla Luana Dinardo, Hadassa Campos Santos, André Ramos Vaquero, André Ricardo Martelini, Luis Alberto Oliveira Dallan, Adriano Mesquita Alencar, José Eduardo Krieger, Alexandre Costa Pereira

**Affiliations:** 1 Laboratory of Genetics and Molecular Cardiology, Heart Institute (InCor), University of São Paulo Medical School, São Paulo, Brazil; 2 Laboratory of Microrheology and Molecular Physiology, Physics Institute, University of São Paulo, São Paulo, Brazil; 3 Coronary Surgery Unit, Heart Institute (InCor), University of São Paulo Medical School, São Paulo, Brazil; Medical University Innsbruck, AUSTRIA

## Abstract

**Aims:**

Recent evidence shows the rigidity of vascular smooth muscle cells (VSMC) contributes to vascular mechanics. Arterial rigidity is an independent cardiovascular risk factor whose associated modifications in VSMC viscoelasticity have never been investigated. This study’s objective was to evaluate if the arterial rigidity risk factors aging, African ancestry, female sex, smoking and diabetes mellitus are associated with VMSC stiffening in an experimental model using a human derived vascular smooth muscle primary cell line repository.

**Methods:**

Eighty patients subjected to coronary artery bypass surgery were enrolled. VSMCs were extracted from internal thoracic artery fragments and mechanically evaluated using Optical Magnetic Twisting Cytometry assay. The obtained mechanical variables were correlated with the clinical variables: age, gender, African ancestry, smoking and diabetes mellitus.

**Results:**

The mechanical variables Gr, G’r and G”r had a normal distribution, demonstrating an inter-individual variability of VSMC viscoelasticity, which has never been reported before. Female sex and smoking were independently associated with VSMC stiffening: Gr (apparent cell stiffness) *p* = 0.022 and *p* = 0.018, R^2^ 0.164; G’r (elastic modulus) *p* = 0.019 and *p* = 0.009, R^2^ 0.184 and G”r (dissipative modulus) *p* = 0.011 and p = 0.66, R^2^ 0.141.

**Conclusion:**

Female sex and smoking are independent predictors of VSMC stiffening. This pro-rigidity effect represents an important element for understanding the vascular rigidity observed in post-menopausal females and smokers, as well as a potential therapeutic target to be explored in the future. There is a significant inter-individual variation of VSMC viscoelasticity, which is slightly modulated by clinical variables and probably relies on molecular factors.

## Introduction

Recent studies have demonstrated VSMC viscoelasticity contributes to the vessels’ overall mechanics, contradicting the previous paradigm which associated vascular compliance only with extracellular matrix composition (ECM) [1]. Under physiological conditions, it has been shown the VSMC mechanical behavior varies according to the position in arterial tree and intensity of regional mechanical forces. As the variations of VSMC mechanical phenotype were in accordance with the variations of vascular compliance, the association between these two variables was suggested [2]. In pathological scenario, VSMC cytoplasm stiffening has already been associated with aging and arterial hypertension, causing arterial rigidity in the absence of ECM modifications [3, 4].

Arterial rigidity is an independent cardiovascular risk factor caused by diverse pathological clinical variables: hyperlipidemia [5], diabetes mellitus [6], arterial systemic hypertension [7] and smoking [8], as well as by some physiological factors: aging, female sex and black race [9, 10]. Proteomics studies show almost one-third of the differences found between stiff and distensible vessels are due to the differential expression of proteins involved in the mechanical regulation of vascular function, especially the components of VSMC cytoskeleton and ECM [11]. Even though this difference exists, the ability of most arterial rigidity risk factors to modify neither VSMC mechanical phenotype nor ECM composition has been properly explored, limiting our understanding of the pathophysiological steps that lead to the reduction of vascular compliance. Exceptionally, hypertension and aging have been associated with VSMCs stiffening and hypertrophy [3, 12], and with VSMCs stiffening [4] and collagen deposit [13], respectively.

The finding that the arterial rigidity risk factors cause VSMC stiffness would not only help in explaining the pathophysiology of the disease, but would also represent a therapeutic option, since the VSMC mechanical phenotype is extremely plastic [14]. Based on what was exposed, the main objective of this study was to evaluate if the arterial rigidity risk factors: aging, African ancestry, female sex, smoking and diabetes mellitus are associated with VMSC stiffening.

## Methods

### Patient recruitment

The study and its consent procedure were approved by our local ethics committee (Comissão de Ética para Análise de Projetos de Pesquisa—CAPPesq USP, number of the project 0272/11). The study was conducted in accordance to the Declaration of Helsinki. All patients included in the study were subjected to coronary artery bypass surgery between 2012 and 2014. The participants were aware of the study objectives and signed the informed consent approved by our local ethics committee. A sample of blood and a fragment of the internal thoracic artery were obtained during the surgical act. All clinical data were obtained using electronic medical records and, in case of dubious information, the patient was contacted for clarification. Estimated glomerular filtration rate (eGFR) was calculated using Cockroft-Gault formula and body mass index (BMI) was calculated using patients’ weight and height measured immediately before the surgery.

### Primary culture (VSMCs isolation)

The chosen experimental set up involved the use of a large repository of primary cell lines mechanically evaluated at low culture passaging using Optical Magnetic Twisting Cytometry assay. This was an attempt to maximize the preservation of VSMCs *in vivo* characteristics, as well as to perform the mechanical measurements without cell detachment from the substrate, keeping the cytoskeleton organization.

**Fig 1:**
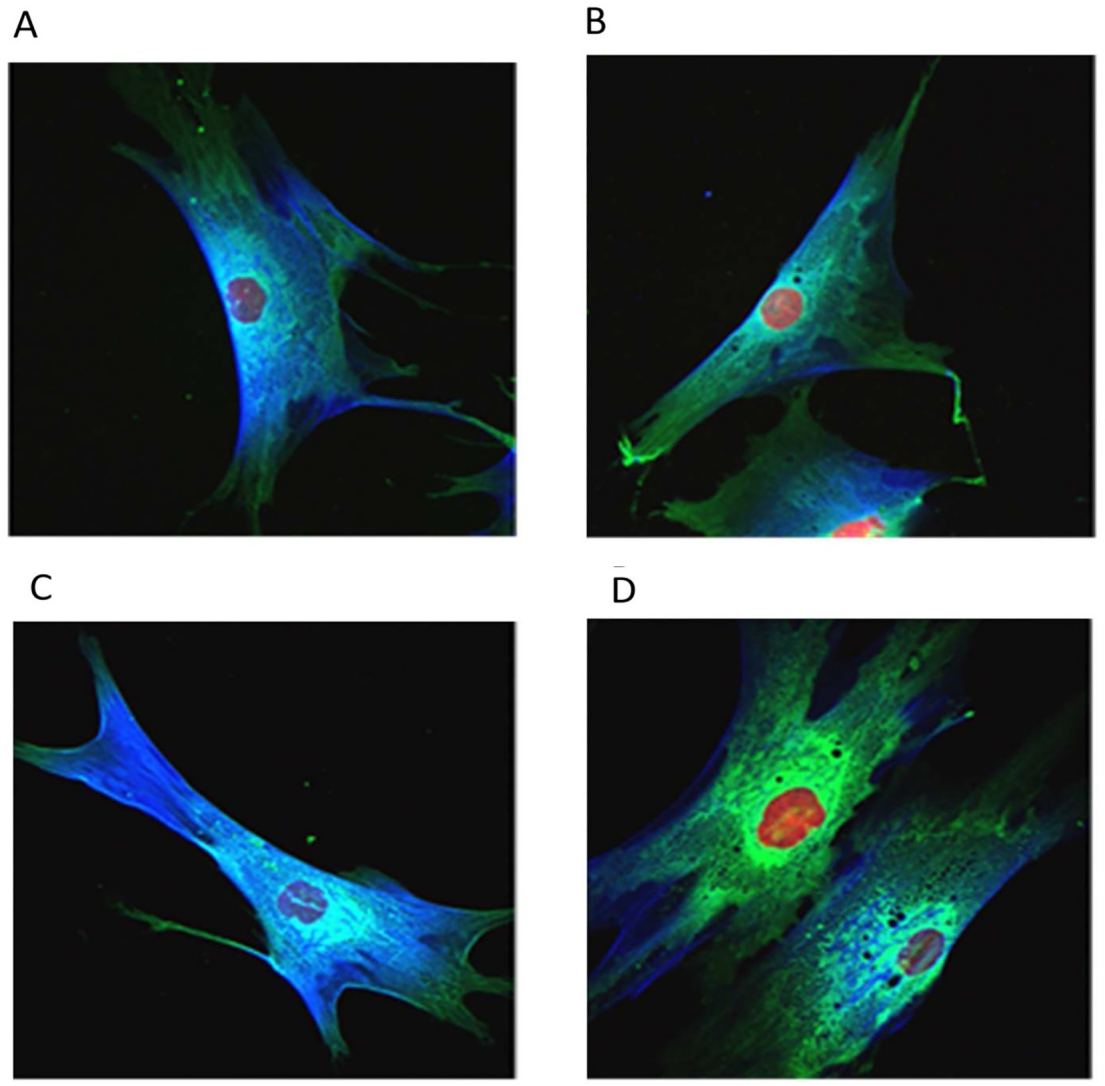
VSMCs characterization. VSMCs obtained using the primary explant technique were characterized using immunofluorescence microscopy with the following primary antibodies: alpha- smooth muscle actin (A2547, Sigma Aldrich®), anti-myosin heavy chain (ab683, Abcam®) and calponin (C6047, Sigma Aldrich®) before the beginning of the experiments. The obtained primary cultures exhibited more than 90% of cells positive for alpha-smooth muscle actin, myosin heavy chain and calponin. (A and B): VSMCs exhibiting positive staining for calponin (blue) and myosin heavy chain (green). Red: nucleus. (C and D): VSMCs exhibiting positive staining for calponin (blue) and alpha- smooth muscle cell actin (green). Red: nucleus. This characterization protocol has a high capability of identifying SMCs amongst possible contaminants.

Internal thoracic artery fragments were used to isolate VSMCs using the primary explant technique. Briefly, the vessel’s lumen was opened and a mechanical friction was applied in order to remove the endothelium. The vessel was then cut into small fragments (approximately 3mm x 3mm), which were placed on a 6-well plastic plate (one or two fragments per well), with the luminal face down. Dulbecco’s Modified Eagle’s Medium with high glucose (Sigma Aldrich®, catalog number D0572) supplemented with 20% fetal bovine serum (Gibco®) and 1% of Penicillin-Streptomycin solution (Sigma-Aldrich®, catalog number P4333) was added to the wells (1cc per well). Isolated VSMCs were characterized using immunofluorescence microscopy with the following primary antibodies: alpha- smooth muscle actin (Sigma Aldrich®, catalog number A2547), anti-myosin heavy chain (Abcam®, catalog number ab683) and calponin (Sigma Aldrich®, catalog number C6047). The obtained primary cultures exhibited more than 90% of cells positive for alpha-smooth muscle actin, myosin heavy chain and calponin (**Fig 1**). When 90% of cellular confluence was achieved in the 6-well plate, cells underwent tripsinization and were seeded on a T25 bottle. There, when 90% of confluence was reached, cells were prepared for the mechanical measurement as it will be described later.

For the mechanical experiments, VSMCs in second culture passage were used, as previous standardized for Optical Magnetic Twisting Cytometry (OMTC) assay [15]. The internal thoracic artery was chosen as object of study since the mechanical properties of its VSMCs, under physiological conditions, are similar to that presented by thoracic aorta VSMCs, as previously demonstrated by our group [2].

### Optical Magnetic Twisting Cytometry (OMTC)

OMTC assay is a method used to measure cell viscoelasticity based on the application of a sinusoidal magnetic torque to ferromagnetic beads, which are intrinsically attached to the cytoskeleton stress fibers [16, 17]. VSMCs (2^nd^ culture passage) were plated on a 96-well plate (Corning®, catalog number 9102) previously coated with 3% porcine gelatin (Sigma Aldrich®, catalog number G9136) at a concentration of 1 x 10^4^ cells per well. Twelve hours after plating, VSMCs were serum-deprived for 24h and, then, RGD-coated ferromagnetic beads were added at a concentration of 10μg per well. VSMCs were then incubated for 20 minutes and washed once to remove loosely bound beads before the performance of mechanical measurements.

Beads were first magnetized horizontally (9,000 Gauss) and, then, a vertical magnetic field oscillating at 0.75 Hz was put into action (90 Gauss). The resulting bead displacement was optically registered by a charge-coupled device camera mounted on an inverted microscope (Leica DMI-4000). The ratio between the Fourier transformation of the specific torque and bead displacement defined a complex apparent stiffness of the cell, g*(f), measured in units of Pa nm^-1^. The apparent stiffness g*(f) was converted into a shear modulus G* after taking into consideration the shape and thickness of the cell and the degree of bead embedding [18]. |G*| at 0.75Hz was labeled G. Based on this value, it was possible to also compute the elastic modulus G’ and the loss modulus *G*” for the cell.

### Genetic ancestry determination

DNA was extracted using QIAamp DNA Mini-kit® and genotyped using Affymetrix GeneChip Human Mapping SNP Array®, according to manufacturer instructions. The evaluation of genomic ancestry was conducted using the Admixture program [19]. Admixture is a software tool for maximum likelihood estimation of individual ancestries from multilocus SNP genotype datasets. Specifically, Admixture uses a block relaxation approach to alternately update allele frequency and ancestry fraction. This software estimates parameter standard errors using bootstrapping. Since the contributions of different ancestral genomes have previously been described by our group, as well as others, we used a supervised approach for ancestry determination [20]. We used 200 bootstrap replicates (default) and k = 3 (number of populations assumed for the analysis). This analysis was done using all 101.348 common autosomal SNPs between the study population and reference. We assumed as reference ancestral populations individuals from the Human Genome Diversity Project (HGDP) [21]: Pima, Maya as Amerindians and from HapMap project [22]: Africans—YRI (Yoruba in Ibadan, Nigeria), LWK (Luhya in Webuye, Kenya), ASW (Americans of African Ancestry in SW, USA); European—CEU (Utah Residents (CEPH) with Northern and Western European ancestry) and TSI (Tuscan in Italia).

### Statistical analysis

Any potential bias due to different bead batches was corrected using a linear regression method and the non-standardized residues (named Gr, G’r and G”r) were used as the variables of comparison between the groups. Univariate analysis was performed using t-Student test for the categorical variables and Pearson correlation test for the numerical variables. Multivariate analysis was done using multiple linear regression, considering as dependent variables: Gr, G’r and G”r and, as independent variables: age, sex, African ancestry, diabetes mellitus, smoking and any clinical variable whose association with the mechanical variables resulted a *p* value of 0.2 or less in the univariate analysis. A *p* value less than 0.05 was considered significant in the multivariate analysis. All statistical tests were held in SPSS software, 18^th^ version.

## Results

**Fig 2:**
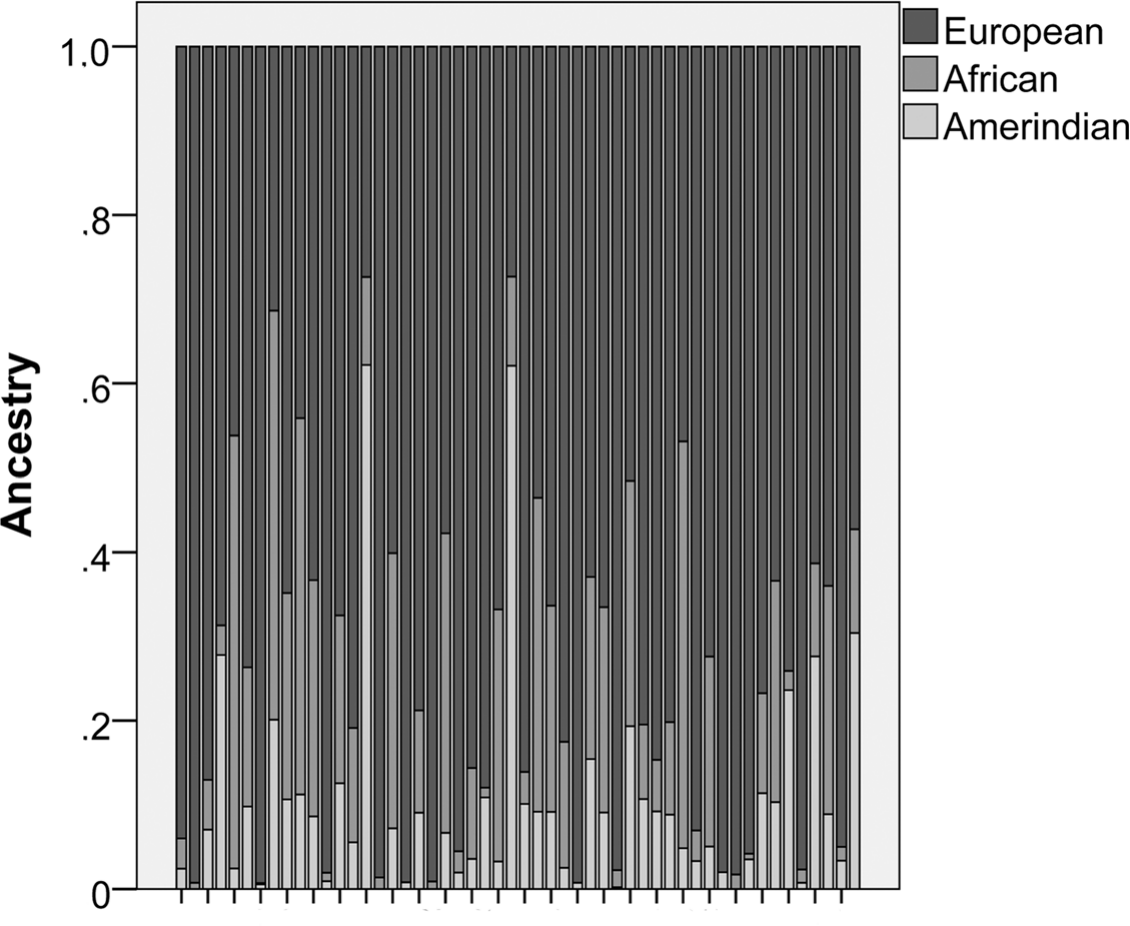
Dispersion of genetic ancestry within the studied population. The graph highlights that all included patients had a predominance of European genetic ancestry in comparison to both African and Amerindian genetic ancestry.

**Fig 3:**
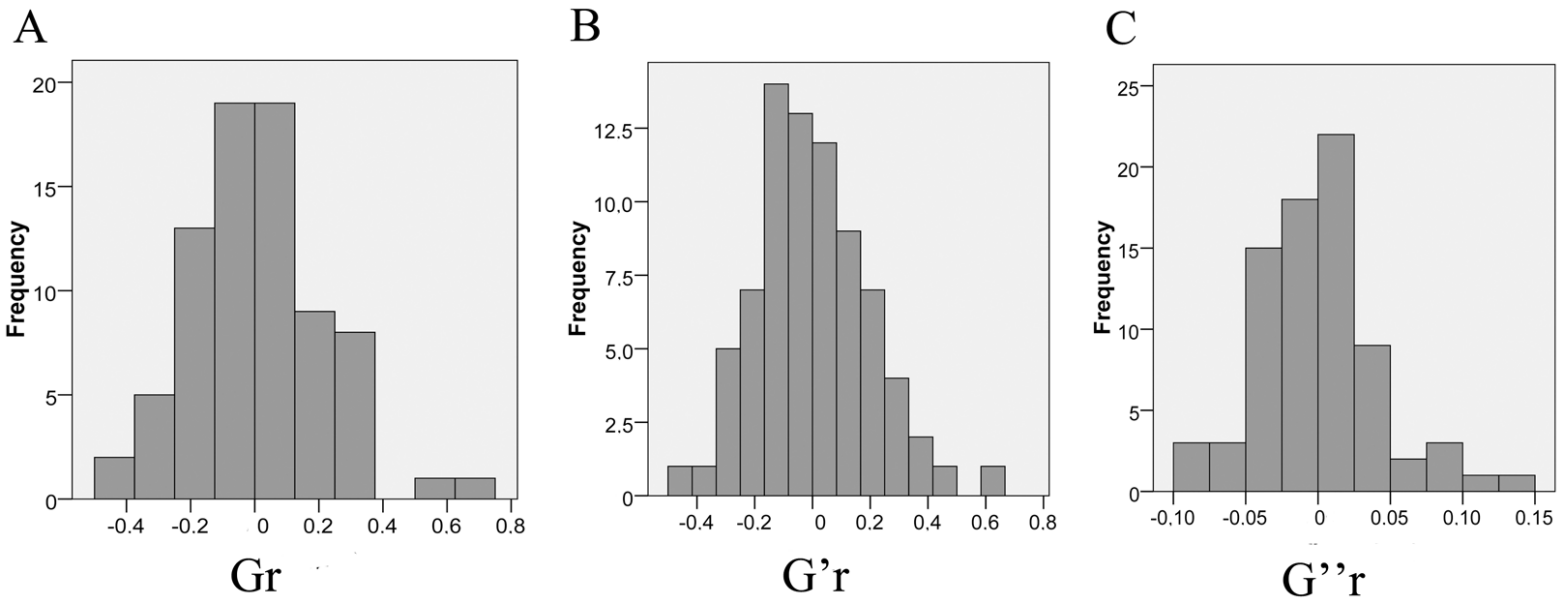
Distribution of the mechanical variables that reflect VSMC viscoelasticity amongst the studied population. The graphs outline that Gr (apparent cell stiffness), G’r (elastic modulus) and G”r (dissipative modulus) present a normal distribution (Kolmogorov-Smirnov test: p = 0.2, p = 0.2 and p = 0.089, respectively) and also highlight the inter-individual variability of this biological trait, never described before.

Eighty patients with chronic coronary heart disease subjected to revascularization surgery were included in the study. The studied population exhibited a predominance of men and European ancestry (**Fig 2**), besides being homogeneous for the presence of systemic hypertension and hyperlipidemia (**Table 1**). All women included in this study were post-menopausal. For each included patient, six replicates of the OMTC experiment were performed and the median value of G, G” and G’ obtained from these assays was used in the statistical analyses. The mechanical variables Gr, G’r and G”r had a normal distribution (Kolmogorov-Smirnov test: p = 0.2, p = 0.2 and p = 0.089, respectively), as exposed on **Fig 3**. This distribution pattern demonstrates an inter-individual variability of VSMC viscoelasticity, which has never been reported before.

**Table 1:** Clinical characteristics of the patients included in the study.

**Number**	80
**Chronic coronary heart disease**	80 (100%)
**Gender** (female/male)	22 (27.5%) / 58 (72.5%)
**Age** (years/mean +/- SD)	63.6 +/- 9.6
**Ancestry** (mean +/- SD)	
European	0.75 +/- 0.2
African	0.15 +/- 0.14
Amerindian	0.1 +/- 0.1
**Diabetes Mellitus** (yes/no)	42 (52.5%) / 38 (47.5%)
**Smoking** (ever/no)*	41 (57%) / 31 (43%)
**Hypertension** (yes/no)*	74 (100%)
**Hyperlipidemia** (yes/no)*	64 (90%) / 7 (10%)
**Chronic Renal Failure** (yes/no)	8 (10%) / 72 (90%)
**Acute coronary syndrome** (ever/no)	55 (68.7%) / 25 (31.3%)
**Symptomatic stable angina** (yes/no)*	38 (50.7%) / 37 (49.3%)
**Chronic obstructive pulmonary disease** (yes/no)	2 (2.5%) / 78 (97.5%)
**eGFR** (mL/min/1.73m^2^) (mean +/- SD)	68.81 +/- 29.16
**BMI** (Kg/m^2^) (mean +/-SD)	28.26 +/- 4.63
**Educational status** *	
Elementary school	34 (43.6%)
Middle school	11 (14.1%)
High school	20 (25.6%)
College	13 (16.7%)
**Medications in use** (yes/no)*	
Angiotensin converting enzyme inhibitor	35 (45%) / 43 (55%)
Beta-blocker	45 (58%) / 33 (42%)
Calcium channel blocker	21 (27%) / 57 (73%)
Lipid-lowering medication	61 (78.2%) / 17 (21.8%)

* In those variables, the total sum of patients is inferior to 80 due to missing data. The presented percentage comprehends only valid cases.

In univariate analysis, the variables Gr and G’r differed between women and men (p = 0.042 and 0.04, respectively) and ever-smokers and non-smokers (p = 0.023 and 0.012, respectively). The other variables of interest (diabetes mellitus, African ancestry, age) were not significantly associated with the studied cellular phenotypes. Also, in univariate analysis, the confounder variables hypertension, hyperlipidemia, chronic renal failure, acute coronary syndrome, symptomatic angina, eGFR, BMI, educational status, medications in use and chronic obstructive pulmonary disease were not significantly associated with any mechanical variables and exhibited a *p* value greater than 0.2. Men and women presented similar rates of diabetes mellitus (p = 0.62). Multivariate analysis was then performed using multiple linear regression including the dependent variables (Gr, G”r and G’r) and the independent variables age, gender, African ancestry, diabetes mellitus and smoking (Table 2). Female sex was found to be a significant predictor of Gr (*p* = 0.022), G’r (*p* = 0.019) and G”r (*p* = 0.011), while smoking was a significant predictor of Gr (*p* = 0.018) and G’r (*p* = 0.009) (**Table 2**). The positive effect of female sex and smoking on the cellular mechanical variables Gr and G’r occurred independently of the variables age, African ancestry and diabetes mellitus.

**Table 2:** Statistical model obtained when evaluating factors capable of modifying VSMC viscoelasticity (multivariate analyses).

**Gr**	**Unstandardized Coefficients**		
**Model**	**β**	**Std. Error**	***p* value**
Constant	-.028	.226	
Gender (1-Male / 2-Female)	.125	.053	.022
Smoking (1-Ever Smoker / 2-Non-smoker)	-.114	.047	.018
Age (years)	.000	.003	.933
African Ancestry (range: 0–1)	.092	.208	.661
Diabetes Mellitus (1-Yes / 2-No)	-.008	.047	.859
R^2^ = .164 (*p* = .042)			
**G’r**	**Unstandardized Coefficients**		
**Model**	**β**	**Std. Error**	***p* value**
Constant	-.089	.211	
Gender (1-Male / 2-Female)	.12	.05	.019
Smoking (1-Ever Smoker / 2-Non-smoker)	-.119	.044	.009
Age (years)	.001	.002	.823
African Ancestry (range: 0–1)	.1	.194	.608
Diabetes Mellitus (1-Yes / 2-No)	.004	.044	.934
R^2^ = .183 (*p* = .023)			
**G”r**	**Unstandardized Coefficients**		
**Model**	**β**	**Std. Error**	***p* value**
Constant	-.009	.047	
Gender (1-Male / 2-Female)	.29	.11	.011
Smoking (1-Ever Smoker / 2-Non-smoker)	-.018	.01	.066
Age (years)	.0	.01	.859
African Ancestry (range: 0–1)	.012	.04	.766
Diabetes Mellitus (1-Yes / 2-No)	.001	.01	.901
R^2^ = .141 (*p* = .082)			

**Fig 4:**
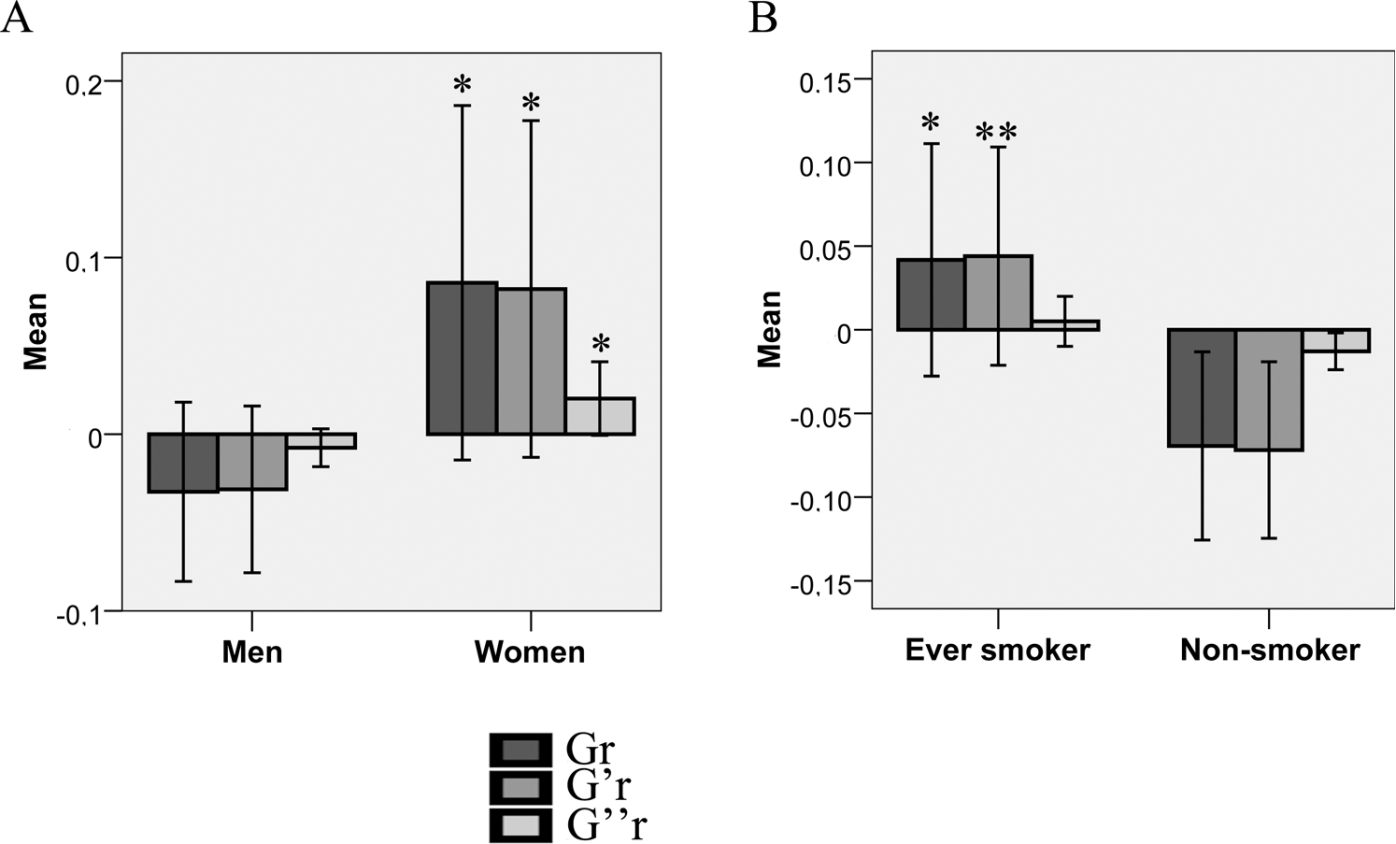
Comparison of VSMC mechanics between men and women / ever smokers and non-smokers. (A) Graph outlining the statistical comparison between men and women regarding VSMC mechanics. Women presented a significant higher apparent VSMC stiffness (Gr) in comparison to men, mainly due to its elastic modulus (G’r). (B) Similarly to what was observed with women, ever smokers presented a significant higher apparent VSMC stiffness (Gr) in comparison to non-smokers, mainly due to its elastic modulus (G’r). Error bars represent 95% CI and their size is compatible with other OMTC experiments in which a per-well analysis was performed. For each patient, 6 replicates of OMTC experiment were performed.

This result demonstrates the influence of the variables female sex and smoking on the increase of VSMC apparent stiffness (G) and its elastic component (G’r), the latter representing cell rigidity (**Fig 4**A and **4**B). Female sex was also associated, in a smaller proportion, to an increase of the mechanical dissipative modulus (G”r), which represents cell viscosity (**Fig 4**A). The other clinical variables of interest (diabetes mellitus, African ancestry and age) were not significantly associated with none of the mechanical variables (**Table 2**). The fact that the resulting multivariate model exhibited a R^2^ of 0.164 (p = 0.042) for Gr, 0.184 (p = 0.023) for G’r and 0.141 (p = 0.082) for G”r is important to highlight. This finding shows that most of observed inter-individual variability of VSMC viscoelasticity (more than 80%) is not explained by our studied clinical variables and probably relies on other modulators, as it will be discussed later.

## Discussion

Our results demonstrate, for the first time, the inter-individual variation of VSMC viscoelasticity and the association between smoking / female sex and VSMC stiffening. The pro-stiffening effect of these clinical variables represents an important element for understanding the vascular rigidity observed in post-menopausal females and smokers, since significant changes in both ECM and VSMC have never been described in these conditions [8, 23]. Also, VSMC stiffening was observed in the absence of endothelium, what is especially relevant in the case of smoking, whose resulting arterial rigidity was always associated only with the dysfunction of endothelial cells [24]. Finally, it was demonstrated most of observed inter-individual variability of VSMC viscoelasticity is not explained by clinical variables of risk for arterial rigidity, probably relying on molecular modulators.

The role played by VSMCs on the vessel mechanics has been shed to light only recently, contradicting the classical concept that associated vascular compliance only with ECM composition [1]. In this regard, there are two experimental situations in which the VSMC cytoplasm rigidity was associated with an increase of vascular tone: arterial hypertension [3] and senile arterial rigidity [4]. In the first situation, it was demonstrated the VSMCs from spontaneously hypertensive rats were stiffer than the VSMCs from non-hypertensive controls, using atomic force microscopy to measure cell rigidity and an engineered aortic tissue model to evaluate the impact of stiffer VSMCs on vascular tone. In the second situation, the VSMCs from aged monkeys with arterial rigidity proved to be stiffer than the VSMCs from young animals using the same previously described methodology. In both studies, no ECM abnormalities were identified.

The present study is the first to assess the viscoelasticity of human primary VSMCs and correlate this biological trait with five important clinical factors of risk for arterial rigidity (age, gender, race, diabetes mellitus and smoking). It was found smoking and female sex are independent predictors of VSMCs stiffness. Considering that there are no studies showing significant changes in ECM and VSMCs of smokers and women, as it will be explored later, this represents the first description of a vascular abnormality of significance associated with these clinical variables. Even though it is difficult to quantify the relative contribution of VSMC mechanics and ECM for the arterial phenotype in these patients, our data suggest VSMC stiffening represents one possible contributory factor for the abnormal mechanical phenotype observed in association with smoking and female sex [3, 4].

It has already been demonstrated post-menopausal women exhibit higher aortic rigidity and lower vascular compliance in relation to men [23], situation associated with a significant impact on their mortality [25] and probably caused by estrogen deprival [26–28]. Even though some authors argue that the smaller diameter of aortic root is responsible for women increased pulse pressure [29], there are strong evidences pointing out that the vascular wall composition is the most important factor responsible for it [23]. Gender differences concerning neither the ECM composition nor the VSMCs were previously evaluated. Our finding that women VSMCs are stiffer irrespective of other clinical variables (age, race, smoking, diabetes, hypertension and hyperlipidemia) represents the first evidence of vascular structural differences between male and female gender and offers a therapeutic possibility to be further evaluated.

The exposure to tobacco is also an independent risk factor for arterial rigidity [30, 31] and the exposure removal is incapable of completely reversing the established vascular mechanical modifications [32]. The abnormal vascular contractility observed in smokers has been always extensively associated with the injury of endothelial cells due to oxidative and inflammatory stress [24, 33]. That means that the physiological response of VSMCs to the dysfunctional endothelium was the only responsible for arterial rigidity [24]. Contradicting this fact, our study shows that smokers’ VMSCs are stiffer than non-smokers’ VSMCs regardless of the presence of the endothelium, suggesting a direct action of smoking on VSMCs or a final scenario of chronic VSMC modifications which could have been once deflagrated by the endothelium or by changes in ECM. The literature is scarce regarding smoking-associated ECM / VSMC abnormalities. There are evidences of increased activity of the matrix metalloproteinases MMP-2 and MMP-9 in association with nicotine exposure [34] and of increased production of PDGF, leading to chronic modifications in VSMC cytoskeleton, following tobacco exposure [35]. However, none of these studies have evaluated if the amount /composition of ECM is altered in smokers, as well as if the VSMC cytoplasm is more rigid in the same patients. Our finding that VMSC mechanics is affected by smoking helps to explain its association with arterial rigidity.

The finding that VSMCs are stiffer in women and in smokers is of great interest because it may represent a future therapeutic option to their associated arterial rigidity, given the plasticity of VSMC mechanical phenotype [14]. ECM composition and regional mechanical forces are factors known to modulate VSMC mechanics, but difficult to be modified *in vivo* [2]. In contrast, there are peptides capable of directly increasing VSMC tone, namely angiotensin 2 [36] and aldosterone [37], both associated with arterial rigidity and whose blockage may ameliorate the vascular compliance. Moreover, drugs that increase the endothelium production of nitric oxide, such as the 3-hydroxi-3-methylglutaryl coenzyme A reductase inhibitor fluvastatin, are also capable of indirectly reducing VSMC tone [38]. The ability of those options in reducing the stiffening of large arteries observed in women and smokers needs to be explored in the future.

Finally, this is the first study to demonstrate the presence of significant inter-individual variability of VSMC viscoelasticity. Even though it was stated that female sex and smoking affect this phenotype, our resulting statistical model was capable of explaining less than 20% of the trait’s variation. Knowing which factors are capable of modulating VSMC mechanics is of great importance, especially considering the growing evidences linking this phenotype with the vessels overall mechanical behavior. Considering this, future studies targeting possible genetic modulators of this trait are warranted as they can provide significant insight into the biology of vascular rigidity.

The present study has some limitations. Even though the variable ancestry was assessed using genetic tools, there was a strong predominance of patients with European origin, hampering the evaluation of a possible influence of the African heritage on VSMC rigidity. Similarly, the dispersion of the variable age within the sample was small, reducing the power of the correlation between this variable and the VSMC viscoelasticity. Even though the association between arterial hypertension and VSMC stiffening has already been demonstrated in the experimental scenario using animal models, in this study, the pro-stiffening effect of this variable could not be evaluated because of its universal presence in the studied sample. Last, the vascular properties of coronary arterial disease patients may vary [39] and, as the pulse wave velocity was not measured in our patients, the direct correlation between VSMC viscoelasticity and arterial rigidity could not be made.

In conclusion, female sex and smoking are independent predictors of VSMC stiffening. The described VSMC rigidity represents a significant change of the vascular media layer of smokers and post-menopausal females, thus becoming an important element in understanding the vascular rigidity observed in this population. Also, considering the plasticity of VSMC mechanical phenotype, this finding is a potential therapeutic target to be explored in the future. There is a significant inter-individual variation of VSMC viscoelasticity, which is slightly modulated by clinical variables and probably relies on molecular factors.
